# Virulence Spectra of Hungarian *Pyrenophora teres* f. *teres* Isolates Collected from Experimental Fields Show Continuous Variation without Specific Isolate × Barley Differential Interactions

**DOI:** 10.3390/jof10030184

**Published:** 2024-02-28

**Authors:** József Bakonyi, Diána Seress, Zoltán Á. Nagy, Ildikó Csorba, Mónika Cséplő, Tibor A. Marton, Anke Martin, Klára Mészáros

**Affiliations:** 1 Plant Protection Institute, HUN-REN Centre for Agricultural Research, Herman Ottó Str. 15, 1022 Budapest, Hungary; seress.diana@atk.hun-ren.hu (D.S.); csorba.ildiko@atk.hun-ren.hu (I.C.); 2 Phytophthora Research Centre, Department of Forest Protection and Wildlife Management, Faculty of Forestry and Wood Technology, Mendel University in Brno, 613 00 Brno, Czech Republic; zoltan.nagy@mendelu.cz; 3 Agricultural Institute, HUN-REN Centre for Agricultural Research, Brunszvik Str. 2, 2462 Martonvasar, Hungary; cseplo.monika@atk.hun-ren.hu (M.C.); meszaros.klara@atk.hun-ren.hu (K.M.); 4 Seed Production Research, Syngenta Hungary Ltd., Alíz Str. 2, 1117 Budapest, Hungary; tibor.marton@syngenta.com; 5 Centre for Crop Health, University of Southern Queensland, Toowoomba, QLD 4350, Australia; anke.martin@unisq.edu.au

**Keywords:** correspondence analysis, GGE biplot, net form net blotch, pathogenic variation, pathotypes

## Abstract

*Pyrenophora teres* f. *teres* (Ptt), the causal agent of net form net blotch (NFNB) disease, is an important and widespread pathogen of barley. This study aimed to quantify and characterize the virulence of Ptt isolates collected from experimental fields of barley in Hungary. Infection responses across 20 barley differentials were obtained from seedling assays of 34 Ptt isolates collected from three Hungarian breeding stations between 2008 and 2018. Twenty-eight Ptt pathotypes were identified. Correspondence analysis followed by hierarchical clustering on the principal components and host-by-pathogen GGE biplots suggested a continuous range of virulence and an absence of specific isolate × barley differential interactions. The isolates were classified into four isolate groups (IG) using agglomerative hierarchical clustering. One IG could be distinguished from other IGs based on avirulence/virulence on one to five barley differentials. Several barley differentials expressed strong resistance against multiple Ptt isolates and may be useful in the development of NFNB-resistant barley cultivars in Hungary. Our results emphasize that the previously developed international barley differential set needs to be improved and adapted to the Hungarian Ptt population. This is the first report on the pathogenic variations of Ptt in Hungary.

## 1. Introduction

*Pyrenophora teres* Drechs. f. *teres* Smed.-Pet. (Ptt), the causal agent of net form net blotch (NFNB) disease [1], is an important and widespread pathogen of barley [2,3]. Typical symptoms initiated by this ascomycete fungus can be recognized based on longitudinal and transverse necrotic streaks forming characteristic net-like leaf lesions, which are often surrounded by a chlorotic halo on sensitive host cultivars [1]. Yield loss due to net blotch is estimated to be 10–40%, with the potential of total loss if susceptible cultivars are planted in a conducive environment [4,5,6,7]. The disease can also lead to a reduction in kernel size, plumpness, and bulk density and negatively affects malting and feed quality [6,8].

Growing tolerant/resistant cultivars is the most economical and environmentally friendly method of managing plant diseases. Host resistance against NFNB in barley is complicated and can be conferred by both quantitative trait loci and specific major genes [3,9,10,11,12,13,14,15,16]. Quantitative resistance is effective against a range of Ptt isolates differing in virulence and is best expressed during the adult growth stages under field conditions [10,17,18]. Qualitative resistance is controlled by major genes specific to certain Ptt pathotypes and is effective in both seedlings and adult plants [19,20]. Major gene resistance to Ptt may follow a gene-for-gene or inverse gene-for-gene model [3] as Ptt produces host-specific toxins [21,22,23], a kind of virulence factor, which is recognized and acts as a necrotrophic effector in toxin-sensitive cultivars [24,25]. Sarpeleh et al. [26] also identified some non-host selective low-molecular weight compounds that induce chlorosis in barley leaves. This indicates that minor virulence factors may also be important in the pathogenesis of the NFNB fungus [3].

Differences in the virulence of Ptt isolates were first reported by Pon [27] and then by Khan and Boyd [28]. Host cultivars may affect the virulence of plant pathogens. To help regional resistance breeding and agricultural production, the virulence and pathotype composition of local Ptt populations need to be determined [29,30,31,32,33,34,35,36,37,38,39,40,41,42,43,44]. An international standard set of barley differential genotypes, some with known genetic backgrounds of NFNB resistance [45,46], have been proposed to classify the reactions of barley to Ptt and characterize the virulence of the fungus.

Being a livestock and human food and industrial raw material, barley is the third most frequently produced cereal in Hungary, with a growing area of 0.25 million hectares (6% of the total arable land) and 1.1–1.5 million tonnes harvested grain per year (https://www.ksh.hu/stadat_files/mez/hu/mez0021.html, accessed on 19 December 2023). Net form net blotch became a very harmful disease of spring barley in the 1980s [47] and was later considered one of the most important biotic agents threatening the production of both spring and winter barley varieties [48,49]. The fungus has also been identified in wheat in Hungary [50]. A survey from 2006 to 2010 confirmed the abundance and high genotypic diversity of Ptt in both commercial and experimental fields of barley and wheat throughout Hungary [51,52].

Breeders often evaluate the cultivar resistance to plant diseases in local experimental fields, where a large number of host plant cultivars and breeding lines with diverse genetic backgrounds and the absence of fungicide treatments are favorable for a diverse pathogen micro-population. Knowledge of the pathogenic variations of the causal agents in such micro-populations is important for successful disease-resistance breeding. Therefore, our main aims were to quantify the virulence of Ptt in experimental fields of barley in Hungary, determine the pathotype diversity, characterize the interaction pattern between Hungarian Ptt isolates and international barley differentials, and investigate the use of barley differentials to discriminate amongst the virulence of local Ptt isolates. In addition, relationships within isolates and within differentials were inspected. Our paper is the first to report on the pathogenic variations of Ptt in Hungary.

## 2. Materials and Methods

### 2.1. Isolate Collection and Isolation

Naturally infected barley leaf samples were collected in mid-May and early June between 2008 and 2018 from experimental fields of three cereal breeding institutes located in different regions of Hungary within distances of 98–162 km from each other (Table S1). The samples were stored in paper bags in the laboratory. To induce sporulation, the samples were placed in a moist chamber in glass Petri-dishes and incubated under cool white light (OSRAM L36W/640, Prolux Ltd., Budapest, Hungary) in a 16 h light/8 h dark cycle at 20/17 °C for 1–3 days. Monoconidial isolates were then produced by transferring single conidia from the leaves onto V8-juice agar in polystyrene Petri dishes [V8A; 177 mL Campbell’s V8-juice, 3 g CaCO_3_, 16 g Bacto agar (Biolab ZRt, Budapest, Hungary), filled up to 1000 mL with distilled water], using a sterile needle and a Leica MZ6 stereomicroscope (Elektro-Optika Ltd., Erd, Hungary) at 300–400× magnification in a laminar air flow cabinet. After 5 to 7 days, approximately 7 mm-diameter mycelial agar blocks were used from the emerged monoconidial cultures to inoculate both the new V8A plates and autoclaved maize leaves (ML) placed onto the surface of 1.5% water agar [53] in 90 mm polystyrene Petri dishes (ca. 15 mL medium per dish). The plates were kept under the same photoperiod and temperature conditions as mentioned above to screen the isolates for sporulation. For long-term storage, each culture was cultivated on a V8A slant for 7–10 days, overlaid with autoclaved mineral oil, and stored at 15 °C in the dark.

### 2.2. DNA Extraction and PCR-Based Identification

All monoconidial isolates were grown in pea broth (140 g of frozen green peas boiled for 10 min, blended, filtered through a cheese-cloth, and the filtrate amended with 1 g L-asparagine, 0.5 g KH_2_PO_4_, 0.25 g MgSO_4_, 1 mg Thiamine, 10 g sucrose and 15 g Bacto agar per 1 L medium, pH 6.5) in steady cultures for 7–10 days at 18–20 °C in the dark. The mycelia were harvested via filtration, washed with deionized water, freeze-dried, and ground in liquid nitrogen. The total genomic DNA was extracted from 20 mg of mycelium powder according to the short extraction protocol of the E.Z.N.A.^®^ Fungal DNA Mini Kit (Omega Bio-tek Inc., Norcross, GA, USA). The DNA quantity and purity were measured using a NanoDrop 1000^®^ spectrophotometer (Thermo Fisher Scientific Inc., Wilmington, DE, USA). The extracted DNA was kept at −20 °C until further use.

The identity of all the monoconidial isolates was confirmed using the 12 specific primer pairs developed by Poudel et al. [54] to distinguish between the *P. teres* forms. The reaction mixtures were prepared in a volume of 15 µL containing 15 ng of DNA, 1× DreamTaq™ Buffer, 5 µM of dNTPs, 0.5 µM of each primer-pair, and 0.1-unit DreamTaq™ DNA Polymerase. The PCR consumables were from Thermo Fisher Scientific Baltics UAB, Vilnius, Lithuania. The amplifications were performed in 96-well PCR microplates (type PCR-96-C, Axygen Inc., Union City, CA, USA) using an Applied Biosystems 2720 Thermal Cycler (Thermo Fisher Scientific Inc., Budapest, Hungary) programmed as follows: initial denaturation at 94 °C for 3 min, 35 cycles of denaturation at 94 °C for 30 s, annealing at 62 °C for 30 s, extension at 72 °C for 20 s, and a final extension at 72 °C for 7 min. The presence or absence of specific PCR products was assessed after electrophoresis at 100 V for ca. 90 min on a 1% agarose gel (SeaKem^®^ LE Agarose, Lonza, Rockland, ME, USA) stained with 0.5 mg/L ethidium bromide and photographed with the ChemiImager4000 UV Light Imaging System (Alpha Innotech Corporation, San Leandro, CA, USA).

### 2.3. Inoculum Production

All monoconidial cultures verified as Ptt via PCRs were screened for sporulation on V8A and/or ML after 10–11 days of growing under the same conditions as described earlier. The conidia were harvested by adding ca. 10 mL of sterile distilled water containing 0.01% Tween 20 to each 90 mm plate and gently agitating the mycelium with a sterile paintbrush. The suspension was filtered through a metal tea filter, and the inoculum concentration was adjusted to 10,000 particles (mostly conidia with some conidiophores) per mL using a hemocytometer. Altogether, 34 Ptt isolates produced conidia in sufficient numbers for virulence testing.

### 2.4. Barley Genotypes

The barley genotypes included in this study were 16 international Ptt differentials (Beecher, Canadian Lake Shore, Corvett, Diamond, Harbin, Harrington, Manchurian, Prior, Skiff, Tifang, C-20019, CI 5791, CI 4207, CI 9819, CI 9825, CI 11458) that have been used in several studies previously [41,43,44,45,46]. Four barley cultivars that we received from local breeders as “resistant” and “susceptible” controls were included in the differential set. These were Sebastian and Sylphid (both resistant) and Botond and MV Initium (both susceptible). Ten seeds of each genotype were sown into 14-cm plastic pots containing a 1:1 mixture of general horticultural soil and peat. The pots were kept in a plant growth room in an 8 h dark/16 h light cycle at 18 and 22 °C. The lighting was provided by 5 OSRAM Fluora T8 L58W/77 (Prolux Ltd., Budapest, Hungary) and 5 Philips Master TL-D Super 80 58W/830 (Pauer-Land Ltd., Budapest, Hungary) light tubes installed at a height of 1 m above the pots.

### 2.5. Inoculation

Twenty seedlings (two pots) per differential were inoculated with each of the thirty-four isolates 14–15 days after sowing when the second leaves were fully developed. The potted seedlings were spaced at random and evenly within a constant-sized area and sprayed with 250 mL of an inoculum suspension (6.25 mL/pot). A homemade atomizer connected with an air compressor providing 1.5 bar pressure of air was used for spraying. Twenty plants of each barley differential were also treated with sterile distilled water containing 0.01% Tween 20. The plants were incubated in darkness for 24 h at 20 ± 1 °C and 100% relative humidity in a plastic box before they were returned to the growth room. The germination of the conidia (and the conidiophores) was tested by incubating a thin layer of the inoculum suspension in a polystyrene Petri dish together with the inoculated plants for 24 h. The germinated and non-germinated particles were visualized under a light microscope (400× magnification).

### 2.6. Scoring of the Infection Responses and Identification of the Pathotypes

The lesion types were assessed 10 days after inoculation on the central portion of the second leaf according to Tekauz’s 10-digit scale [55]. Symptomless infection responses were given a 0. The infection responses (IRs) were averaged for the 20 inoculated seedlings of each differential (Table S1). Avirulence and virulence were distinguished according to Fowler et al. [41], Gamba et al. [43], Tekauz [56], and Liu et al. [57] by IR scores < 5.0 and ≥5.0 and coded binomially with 0 and 1, respectively. The combination of 0 and 1 values obtained on the 20 barley differentials defined the pathotypes.

### 2.7. Data Analysis

R version 4.3.0. [58] running in RStudio 2023.03.1 + 446 [59] was used for the data analyses. To quantify the virulence of the individual isolates, their virulence frequency profiles were investigated following the Jebbouj and El Yousfi [37] protocol. The IR scores were grouped into one of the following classes: avirulent-1 (avr1) = 0–2.49, avirulent-2 (avr2) = 2.50–4.99, virulent (vir) = 5.00–7.49, and highly virulent (hvir) = 7.50–10. Lack of independence between isolates (row categories) and avirulence/virulence classes (column categories) was tested using Pearson’s chi-squared (Χ^2^) test using the ‘chisq.test()’ function in R. A correspondence analysis (CA) [60] was applied to the frequency data to find the optimal scores for the rows and columns on a small number of dimensions that accounted for the largest proportion of the Χ^2^ value. The similarities in the frequency profiles of the isolates over avirulence/virulence classes and vice versa, as well as the association between the isolates and avirulence/virulence classes, were visually depicted on a symmetric biplot in which both isolates (marked with dots) and avirulence/virulence classes (triangles) were displayed according to their principal coordinates in the first two dimensions. The distance between any two dots or between any two triangles approximated the similarity (or dissimilarity) of the respective isolates or avirulence/virulence classes. The magnitude and direction of the isolate–avirulence/virulence correlations were estimated based on the cosine of the angle between a class vector line (or its extension through the origin) and an imaginary line drawn from the origin to an isolate point (i.e., 0° = perfect positive correlation, 90° and 270° = no correlation, 180° = perfect negative correlation), whereas the cutpoint of a perpendicular line from an isolate point to any avirulence/virulence class vector line (or its extension) approximated the impact of the isolate on that particular avirulence/virulence class [61,62]. To group the isolates based on their avirulence/virulence frequency profiles, the CA was complemented with an agglomerative hierarchical classification procedure on the principal components (HCPC) using all three dimensions from the CA output to utilize 100% of the variability. The pairwise distances between the isolates were measured with the squared Euclidean distance [63,64], and clustering was performed with the WardD1 method [65]. The optimal number of HCPC clusters was determined according to the loss in between-group inertia. The R package ‘Factoshiny’ v.2.4 [66] was used for the CA and HCPC analyses.

Isolates’ general virulence and specificity to differentials, isolate × differential interaction pattern, and the discriminativeness power of differentials amongst the virulence of Ptt isolates were graphically investigated using GGE biplots adopted for host-by-pathogen data [67,68]. The isolates were taken as genotypes (G), and the barley differentials were taken as environments (E). The GGE model included the isolate’s main effect (G) and the isolate × differential interaction (GE). For clarity, the rows and columns of the GGE input data table are hereinafter referred to as entries (isolates) and testers (barley differentials), respectively. R packages ‘GGEBiplots’ v. 0.1.1 [69] and ‘metan’ v. 1.18.0 [70] were used to draw the GGE biplots.

To classify the Ptt isolates and barley differentials, the isolate × barley differential IR score matrix was used without data transformation, centering, and scaling for calculating the unsquared Euclidean distances and hierarchical clustering with the WardD2 method. The R package ‘ClustVis’ v. 0.0.0.9 [71] was applied to draw the dendrograms and the associated heat map.

## 3. Results

### 3.1. Virulence Quantification

Average IR scores ranged between 0 (symptomless) and 10 (total leaf tissue collapse) (Table S1). Only isolate M1 initiated symptomless reactions on two differentials (CI 11458 and Sebastian). In contrast, maximum IR scores were depicted by Ka8 and M12 on MV Initium as well as by Ko3 on Diamond. The average IR scores of the individual isolates across all differentials ranged from 2.17 (M1) to 7.89 (Ko3), with a mean of 4.96 ± 2.41 (Table 1).

Considering 5.00 as a cut-off point between avirulence and virulence, the isolates were virulent in almost half of all the possible combinations (44%, 302 out of 680 cases), and the individual isolates were virulent on at least 2 and up to 20 differentials with an average of 8.88 ± 5.54 differentials. M1 from Martonvásár, Ko1 from Kompolt, and Ka2 and Ka3 from Karcag were virulent to two differentials, whereas Ka14 from Karcag and Ko3 from Kompolt were virulent to the entire differential set (Table 1 and Table S1).

**Figure 1:**
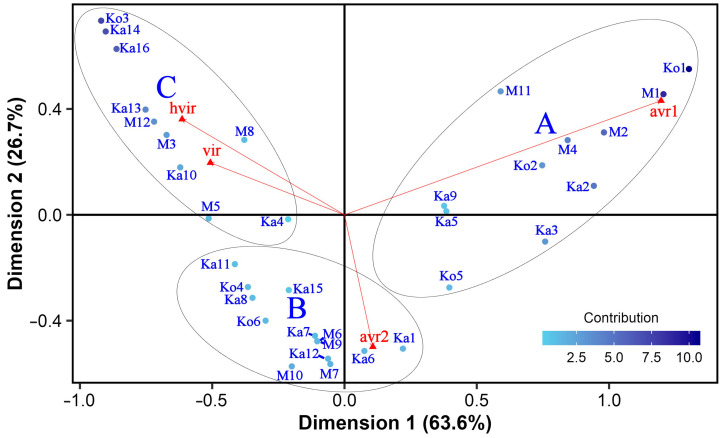
Symmetric biplot of the correspondence analysis based on the frequency profiles of *Pyrenophora teres* f. *teres* isolates (blue dots) in four avirulence/virulence classes (red triangles). The M6 and M9 isolates are represented with a single dot as they have identical frequency profiles. The color scale for the isolates and the length of avirulence/virulence class vectors are proportional to the variance contributing to the plane. HCPC clusters (A, B, and C) are marked by ellipses.

The analysis of frequency data indicated a clear relationship between the isolates and the avirulence/virulence classes according to Pearson’s chi-squared test (Χ^2^ = 412.22, df = 99, *p* < 0.01), and the correspondence analysis identified three dimensions explaining all the variability of the contingency table. The first and second dimensions accounted for 63.6% and 26.7% of the total variability, respectively (Figure 1). Since the sum of these two values was greater (90.3%) than the reference value of 83.8%, which is the 0.95-quantile of the inertia percentages distribution obtained by simulating 1562 data tables of equivalent size on the basis of a uniform distribution, the variability explained by Figure 1 is significant. As shown by the direction of vector lines for the column categories, avirulence was unambiguously separated from and negatively correlated with virulence on this 2-dimensional plane. Dimension 1 opposed class ‘avr1’ to the two virulence classes, whereas dimensions 1 and 2 together separated ‘avr2’ from the other three classes. The vector lines of the two avirulence classes are very close to orthogonal, which indicates almost zero correlation. In contrast, the 2 virulence classes are almost perfectly and positively correlated. Considering the length of vectors, ‘avr1’ was the most variable, whereas ‘vir’ showed the lowest variance.

Several isolate–avirulence/virulence class associations were explored in Figure 1. For instance, isolates in the upper right quarter of the plot were associated with class ‘avr1’. Ko1, Ko2, M1, M2, and M4 are positioned close to the vector line of this class. Thus, they had very strong positive correlations with it. These isolates, especially Ko1 and M1, also considerably contributed to the variance. A single isolate, Ka6, showed a very strong positive correlation and close association with ‘avr2’, whereas some others (Ka1, Ka7, Ka12, M6, M7, M9, and M10) were more loosely connected with it. These isolates had a weak contribution to the variability. The eight isolates in the upper left quarter of the CA map were the most virulent ones. Among these, Ko3, Ka14, and Ka16 contributed most significantly to dimension 1 and the CA map by having the highest frequencies in the two virulence classes (Table 1).

The hierarchical cluster analysis using all three CA dimensions identified three loose HCPC clusters (Figure 1). Each HCPC cluster comprised isolates from all three experimental sites. Eleven isolates with 81% avirulent infections within the cluster (13 to 18 per isolate, on average 16.1; Table 1 and Table S1) and a close relationship with the avirulence classes formed HCPC cluster A. All members of this group were avirulent to eight of the differentials (Beecher, Manchurian, Prior, Tifang, CI 4207, CI 5791, CI 9825, CI 11458) and virulent on Botond and MV Initium. HCPC cluster B contained 13 isolates, out of which M6 and M9 showed the same avirulence/virulence frequency profile. The common feature of this HCPC cluster is avirulence on CI 11458 and virulence on Botond and MV Initium. Eight isolates of HCPC cluster B (Ka1, Ka6, Ka7, Ka12, M6, M7, M9, and M10) also showed a close association with avirulence as they had 13 to 16 (on average 13.8) avirulent infections per isolate (Table 1), whereas the remaining five isolates in HCPC cluster B (Ko4, Ka8, Ka11, Ka15, and Ko6) were avirulent or virulent to equal or almost equal number of barley differentials. In total, the isolates within HCPC cluster B were virulent in 37% of cases (Table 1 and Table S1). HCPC cluster C possessed 10 isolates, which were virulent on at least 11 (Ka4) and up to all (Ka14, Ko3) barley differentials (on average 16.2) in 81% of cases (Table 1 and Table S1), and all were virulent on Botond, Diamond, Tifang, CI 9819 and MV Initium, rendering this cluster the most virulent.

### 3.2. Pathotypes

The 34 Ptt isolates were sorted into 28 distinct pathotypes (PT), of which four were represented by more than one isolate (Table 1 and Table 2). Ka2 from KG Apavár and Ka3 from KH Tas (both collected in Karcag), Ko1 from MV Initium (Kompolt), and M1 from KH Turul (Martonvásár) belonged to PT2. Two isolates, Ka14 from MV Initium (Karcag) and Ko3 from KG Apavár (Kompolt), formed PT13. Ko2 from KG Apavár (Karcag) and M4 from KG Konta (Martonvásár) represented PT16, whereas both M9 from Siberia and M11 from Boreale (both from Martonvásár) belonged to pathotype group 26.

The number of pathotypes observed to be virulent on a particular differential ranged between 5 and 28 (Table 2), with an average value of 13.25. For instance, CI 9825 was only susceptible in the presence of pathotype 2, 13–15, and 25 isolates. In contrast, Botond and MV Initium, which were bred and cultivated in Hungary, were highly susceptible, with IR scores ≥7.5 to the majority of isolates (85.3% and 88.2%, respectively). In fact, all the isolates were virulent on these two cultivars, with IR scores ranging between 5.90 and 9.90 (average of 8.39 ± 1.13) for Botond and between 6.60 and 10 (average of 8.75 ± 0.92) for MV Initium (Table S1).

The three sampling sites looked similar to one another in terms of both the average IR scores (5.14 ± 2.29 for Karcag, 4.79 ± 2.58 for Kompolt and 4.79 ± 2.47 for Martonvásár) and number of virulent reactions per isolate (9.44 ± 5.85 for Karcag, 8.17 ± 6.59 for Kompolt and 8.50 ± 4.98 for Martonvásár) (Table 1). The pathotype diversity was high at each sampling location. The 16 isolates from Karcag represented 15 PTs, each isolate from Kompolt belonged to different pathotypes, and the 12 isolates from Martonvásár were grouped into 11 PTs (Table 1).

### 3.3. General Virulence and Specificity of the Isolates and Isolate × Differential Interactions

**Figure 2:**
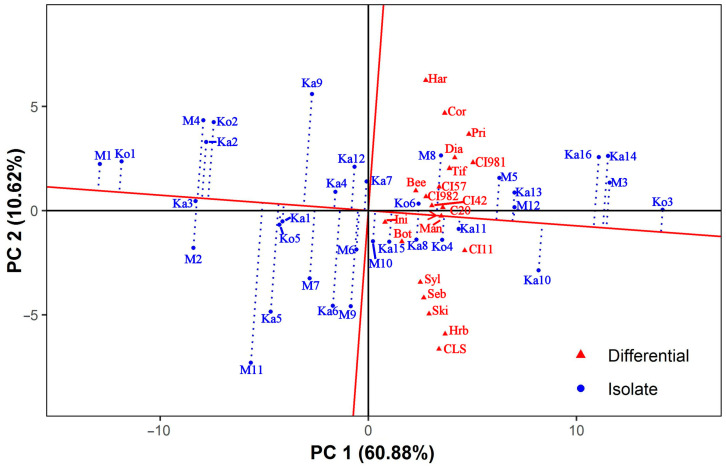
Isolate-focused mean vs. stability GGE biplot showing the general virulence of *Pyrenophora teres* f. *teres* isolates and their specificity to the differentials. The biplot was drawn with the following settings: scaling = no, centering = tester, SVP = entry-focused. Bee = Beecher, Bot = Botond, C20 = C-20019, CI11 = CI 11458, CI42 = CI 4207, CI57 = CI 5791, CI981 = CI 9819, CI982 = CI 9825, CLS = Canadian Lake Shore, Cor = Corvett, Dia = Diamond, Har = Harrington, Hrb = Harbin, Ini = MV Initium, Man = Manchurian, Pri = Prior, Seb = Sebastian, Ski = Skiff, Syl = Sylphid, Tif = Tifang.

The isolate-focused mean vs. stability GGE biplot (Figure 2) facilitates comparison among both the isolates’ general (mean) virulence and specificity to differentials (stability). Since the singular values were entirely partitioned into the isolates’ eigenvectors (entry-focused SVP), this biplot is accurate for comparing isolates [67]. In this type of GGE biplot, the line passing through the origin (mean general virulence of all isolates) and the imaginary average tester, located at the tip of the red arrowhead, is the *x*-axis of the average tester coordinate (ATC), whereas the line perpendicular to it is the ATC *y*-axis. The average tester is defined by the mean PC1 and PC2 scores across all differentials. PC1 and PC2 of this and all other types of isolate-focused GGE biplots presented in this study accounted for 60.88% and 10.62% of the total variation, respectively. The arrowhead pointing to the imaginary average tester indicates the direction of increasing virulence. The GE interaction is proportional to the length of the dotted projection lines.

The isolates showed a scattered distribution, and no pattern related to the geographical origin of the isolates was discovered. Isolate Ko3, being virulent on each differential with the highest average IR score of 7.89, showed the highest general virulence and was also very stable. The next few isolates with high general virulence (M3, Ka14, and Ka16) were slightly variable in their instability, although they had similar average IR scores (7.33 to 7.44) and were virulent to a large number of differentials (15, 20, and 19, respectively). The remaining 11 isolates with positive PC1 coordinates in Figure 2 were virulent to at least 7 (M10) and up to 18 (Ka13) differentials, with average IR scores ranging from 5.15 (M10) to 6.54 (Ka10). The M1, Ko1, Ka3, M2, M4, Ka2, and Ko2 isolates on the left side of the mean vs. stability GGE biplot had average IR scores between 2.17 to 3.41. All of these were virulent to the susceptible control varieties Botond and MV Initium, one (M2) was also virulent to Harbin, and two (Ko2, M4) to both Corvett and Harrington (Table S1). The remaining isolates with negative PC1 coordinates in Figure 2 had average IR scores between 3.88 (M11) and 4.94 (M6) and were virulent to 4 (Ka1 and Ko5) and up to 11 (Ka4) barley genotypes. In general, the 19 isolates with below-average general virulence were less stable than those having above-average general virulence. The least stable isolate was M11, followed by Ka9 and Ka5. In contrast, Ka3 was the most consistent, followed by several other isolates sitting close to the ATC *x*-axis.

**Figure 3:**
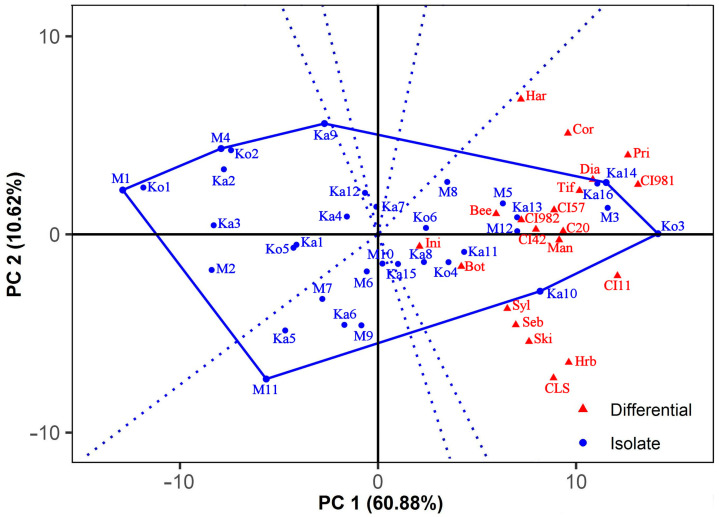
Which-won-where GGE biplot showing the interaction pattern of *Pyrenophora teres* f. *teres* isolates with barley differentials. The biplot was drawn with the following settings: scaling = no, centering = tester, SVP = dual metric. See Figure 2 for the full names of the barley lines.

The interaction pattern of the isolates with barley genotypes is shown in the which-won-where GGE biplot (Figure 3) that was constructed using dual-metric SVP. This SVP option provides a higher goodness-of-fit and a better representation of both the isolates and differentials at the same time than entry or tester-focused SVP methods [72,73]. Seven isolates (Ko3, Ka10, M11, M1, M4, Ka9, and Ka14) formed a polygon that contained all the other isolates. According to the general rule, these vertex isolates were the most outstanding ones; they were either the most or the least virulent to all or certain barley differentials. The lines perpendicular to each side of the polygon divided the biplot into seven sectors. All the differentials fell in a sector for which Ko3 gave the farthest corner.

The isolates in sectors without host genotypes were less virulent, especially the vertex isolates M1 and M11, which were not the winners on any differential. Moreover, they were the least virulent to all or some barley lines.

### 3.4. Discrimitiveness of the Barley Differentials

**Figure 4:**
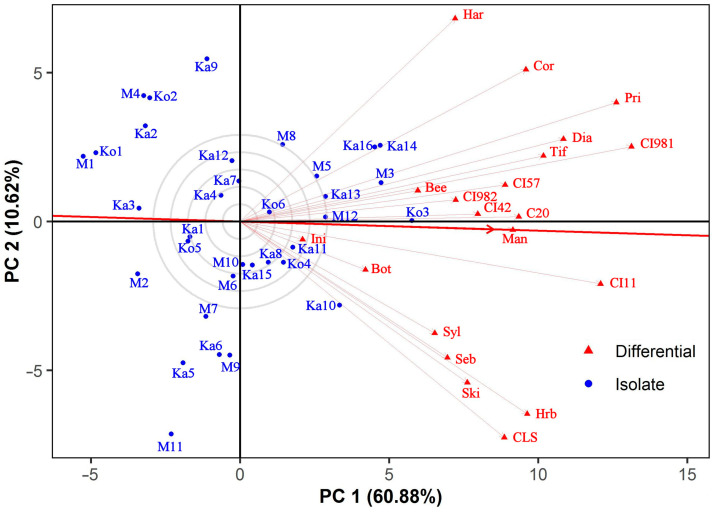
GGE biplot showing the ability of barley differentials to discriminate amongst *Pyrenophora teres* f. *teres* isolates in terms of virulence. The biplot was drawn using the following settings: scaling = no, centering = tester, SVP = tester-focused. See Figure 2 for full names of barley lines.

The ability of barley genotypes to discriminate amongst the virulence of individual Ptt isolates is shown in Figure 4. Similarly to the mean vs. stability GGE biplot, the average-tester axis connects the biplot origin and the imaginary average tester at the tip of the red arrowhead. The vector length of a barley genotype is proportional to its discriminating power, whereas the cosine of the angle between two differentials approximates their correlation [68]. CI 9819 and Prior were equally the most discriminating differentials, followed by CI 11458, Harbin, Canadian Lake Shore, Corvett, and Harrington. In contrast, both MV Initium and Botond behaved almost uniformly, and together with five other testers (Sylphid, Sebastian, Beecher, CI 9825, and CI 4207), they were less discriminating than the average to which Manchurian was the nearest. No differential with outstanding discriminating ability was found. All the host genotypes correlated positively (vector angles < 90°). Canadian Lake Shore and Harrington were the least associated and almost independent genotypes. Canadian Lake Shore, Harbin, Sebastian, Skiff, and Sylphid showed a relatively close correlation as a slightly separate group.

### 3.5. Classification of Ptt Isolates and Barley Differentials

**Figure 5:**
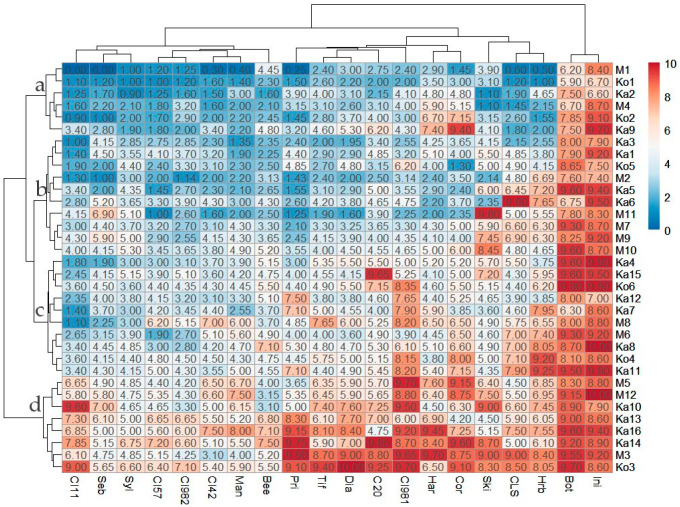
Heat map and clustering of both the *Pyrenophora teres* f. *teres* isolates and barley differentials using the infection response scores (color scale). Hierarchical clustering was performed with the WardD2 method and unsquared Euclidean distances. The isolate groups (a, b, c, and d) are marked at the corresponding nodes. See Figure 2 for the full names of the barley lines.

Four main isolate groups (IG) were identified by hierarchical cluster analysis using the infection response scores as input data (Figure 5). At this level of classification, the maximum within-group variations (distances) were similar. Each IG harbored isolates from each sampling site. Six isolates (Ka2, Ka9, Ko1, Ko2, M1, M4) with a high frequency of avirulent reactions (14 to 18 per isolate, on average 16.6) and the lowest mean within-group IR score of 3.08 across all barley lines (isolate mean 2.17 to 4.39) (Table 1) formed IG ‘a’. All members of this group were avirulent to 14 differentials (Beecher, Canadian Lake Shore, Harbin, Manchurian, Prior, Sebastian, Skiff, Sylphid, Tifang, CI 4207, CI 5791, CI 9819, CI 9825, CI 11458) and virulent on Botond and MV Initium. IG ‘b’ comprised 10 isolates (Ka1, Ka3, Ka5, Ka6, Ko5, M2, M7, M9, M10, M11) virulent on 2 to 7 (on average 5.10) differentials, with a higher mean within-group IR score across all barley lines (IG mean 4.15, isolate mean 3.09 to 5.15) (Table 1). The common feature of isolates in IG ‘b’ is avirulence on 8 barley lines (Diamond, Manchurian, Prior, Tifang, CI 4207, CI 5791, CI 9825, CI 11458) and virulence on Botond and MV Initium. IG ’c’ also contained 10 isolates (Ka4, Ka7, Ka8, Ka11, Ka12, Ka15, Ko4, Ko6, M6, M8). These were virulent on 6 to 14 (on average 9.40) differentials (Table 1). All of these were avirulent on two barley lines (CI 11458 and Sebastian) and virulent on Botond and MV Initium. Members of IG ’c’ had a slightly higher mean within-group IR score of 5.28 (4.67 to 5.88) across all barley lines than the cut-off value between avirulence and virulence in this study (Table 1). IG ‘d’ included the 8 most virulent isolates, which were virulent on at least 13 (M5) and up to all (Ka14, Ko3) barley differentials (on average 17.1) (Table 1), and each isolate was virulent on Botond, Diamond, Harbin, Tifang, CI 9819, CI 11458 and MV Initium. The mean within-group IR score for IG ‘d’ was 6.97 (isolate mean 6.26 to 7.89) across all the barley lines (Table 1).

Associations between IGs and pathotype groups (pathotypes with ≥2 isolates) were not evident. The isolates of PT2 and P16 were grouped in IG ‘a’, except for isolate Ka3, which fell into IG ‘b’ together with the isolates of PT26. The isolates of PT13 were clustered in IG ‘d’ (Table 1).

In terms of avirulence/virulence, only IG ‘d’ could be differentiated from the other IGs. Virulence on CI 11458 was characteristic of IG ‘d’, Tifang differentiated IG ‘d’ from both IGs ‘a’ and ‘b’, and Diamond, Harbin and CI 9819 separated IG ‘d’ from IGs ‘b’, ‘a’ and ‘a’, respectively (Table 3).

The mean IR scores measured for individual barley genotypes (Table S1) ranged between 3.54 (CI 11548) and 8.76 (MV Initium). There are three main groups of barley differentials in Figure 5. The most resistant eight genotypes, CI 11458, CI 5791, CI 9825, CI 4207, Sebastian, Sylphid, Manchurian, and Beecher, form a cluster with average IR scores ≤ 4.15. The moderately susceptible Prior, Tifang, Diamond, C-20019, Canadian Lake Shore, Harrington, Skiff, Corvett, Harbin, and CI 9819 constitute a second cluster (mean IR scores of 4.47–5.82). Botond and MV Initium, the two most susceptible varieties with mean IR scores of 8.4 and 8.76, respectively, reside in the third cluster. There were no barley differential resistant to all the tested isolates.

## 4. Discussion

Studying pathogenic variation in populations of plant pathogens has important implications for successful resistance breeding programs [74]. *Pyrenophora teres* f. *teres*, the causal agent of NFNB is a harmful pathogen of barley in many parts of the world, including Hungary. Since diversity in virulence of this fungus exists on both a local and global level [3], we aimed to investigate the pathogenic variation of Ptt from naturally infected barley plots at Hungarian barley breeding stations.

Seventy percent of our isolates had unique virulence phenotypes, and only four pathotypes had more than one isolate. The most frequent pathotype (PT2), represented by four isolates, occurred at each breeding station. These isolates were virulent on the Hungarian commercial barley cultivars Botond and MV Initium, which were also susceptible to all the other isolates. In contrast, the two isolates of PT13 were virulent on all the differentials. In total, the isolates were virulent in almost half of the cases, and no barley line was resistant to all the isolates. Interestingly, all of the differentials, which have not been grown commercially in Hungary, were susceptible to several of our isolates. Even line CI 5791, which until recently was known to be a universally resistant line [75], was susceptible to some of the isolates.

Tekauz, who introduced the term “pathotype” for Ptt to group isolates based on their virulence phenotypes on a series of nine barley genotypes, observed 45 PTs in 182 western Canadian isolates [56]. A number of studies followed Tekauz’s principle to investigate the pathogenic diversity of *P. teres* f. *teres*. For example, Steffenson and Webster [29] found that 4 out of 13 PTs were present in two-thirds of 91 Californian isolates collected in the major cereal-producing regions and tested on 22 differentials. By extending the same 22 differentials with 3 more, Wu et al. [35] detected 15 PTs in a collection of 23 isolates from 12 barley-growing regions of the world (mainly in North America). With a similar differential set, 49 PTs were observed among 75 isolates from two experimental stations sampled over a 4-year period in North Dakota [57]. Jonsson et al. [76] applied 18 host genotypes, which separated 25 Swedish and two Canadian Ptt isolates from both experimental and commercial fields into 14 PTs, three of which comprised 59% of the isolates. In New Zealand, 11 PTs of 29 isolates derived from barley crops and field trials over three growing seasons were recognized with 31 barley cultivars [33]. Using up to 17 barley genotypes, out of which 13 were also used in our study, Afanasenko et al. [46] discovered 216 PTs in a global collection of 1059 isolates from Australia, Canada, Europe, and Russia. In Algeria, 48 isolates sampled from commercial barley fields in three consecutive years formed 12 PTs [38]. In Turkey, a countrywide survey revealed 24 PTs of 40 isolates on 34 barley genotypes [42]. Douiyssi et al. [30] tested 15 Moroccan isolates from both commercial and experimental fields on 38 barley lines and found that all of them were different. Similarly, no identical virulence phenotypes were seen in Uruguay [43,77]. More recently, 19 PTs were differentiated on 16 barley lines (12 in common with our study) among 20 isolates from seven locations in Iceland [44]. It seems that the high diversity of virulence in our collection is rather similar to the situation in Morocco, Uruguay, and Iceland. *P. teres* is capable of reproducing asexually and sexually when two fungal isolates of different mating types interact [78]. Sexual outcrossing may generate new pathogen genotypes with increased genetic diversity and rapid adaptability to host genotypes with different levels of disease resistance [79]. It was demonstrated that the Hungarian Ptt population is genetically diverse, and both mating types are common in both commercial and experimental fields [51,52]. Perhaps sexual reproduction and the diverse collections of breeding lines/cultivars with variable resistance against Ptt have contributed to a diverse pathogen micro-population at the sampling sites.

Hierarchical clustering following correspondence analysis identified three loose isolate groups close to each other, indicating that virulences of Ptt isolates varied continuously. In general, HCPC clusters represented isolates’ virulence well and were characterized by frequent avirulence and moderate or high virulence. The isolates of the same pathotype fell into the same HCPC clusters, except for PT26, which was shared between two clusters. Additionally, all six isolates of PTs 2 and 14 appeared in a cluster. Previously, Jebbouj and El Yousfi [37] also sorted Moroccan Ptt isolates into distinct HCPC groups, and the pathotypes also did not seem to cluster into separate groups. However, the comparison between our and the Moroccan study is not adequate since the Moroccan pathotypes were identified based on a model analysis.

The scattered distribution of the isolates and the lack of compact isolate clusters in our GGE biplots also suggest a continuous range of virulences. Although there were differences in the isolates’ stability, none showed outstanding instability relative to the others and, at the same time, exceptionally high virulence to any of the differentials. On average, a single isolate was the most virulent across all the differentials. This type of GGE pattern is characteristic of a pathogen population lacking specific isolate—host cultivar adaptation [67]. Continuous variation in Ptt virulence was observed in Morocco, but in contrast to our results, a specific resistance was found against each tested isolate [30]. In Uruguay, neither different virulence groups nor barley genotypes with differential resistance were identified by Gamba and Tekauz [77]. However, a robust study revealed a few isolate-specific interactions besides the predominantly qualitative variation [43]. Arabi et al. [34] found a continuous range of responses on 11 barley genotypes inoculated with Syrian and French Ptt isolates, but the cluster analysis indicated that the isolates exhibited distinct differential virulence patterns, and they clustered into five groups. Surveying commercial barley fields in diverse geographical regions and expanding our differential set could help to better understand the pathogenic behavior of Ptt in Hungary.

The absence of a negative correlation between testers in the GGE biplot analysis is an indicator of the lack of a specific isolate × differential interaction [68]. All barley lines tested by us correlated positively. This further confirms our theory that no specific adaptation exists in the fungal isolate and host plant genotype collections we tested. Prior and CI 9819 showed the best differentiating power among the virulence of individual Ptt isolates. Both exhibited susceptibility to certain isolates from each sampling site. Prior has been considered to be moderately resistant with significant differentiating ability amongst net blotch isolates collected from different regions. No Ptt isolates virulent on this cultivar were found in the Ural region of Russia and The Netherlands, unlike the Czech Republic, Germany, Finland, and Sweden, where 21 to 40 percent of isolates were virulent on Prior [46]. Observations of the Hungarian Ptt isolates (32% virulence on Prior) in our study seem to be comparable to earlier European data. Specificity towards Prior was found in Australia, where it was widely produced until the 1970s but more recent Ptt isolates still exhibited Prior virulence [32,41,80]. A possible explanation for the absence of specific virulence to Prior in our Ptt collection may be that this cultivar has not been commercialized in Hungary. In previous studies, an Ethiopian line, CI 9819, was generally highly resistant without or with low-frequency susceptible reactions to Ptt [33,35,36,38,40,46,81]. Even in Slovakia, a neighboring country with Hungary, virulent infections on CI 9819 were uncommon in spite of the high Ptt pathotype diversity [82]. In the Czech Republic, 17% of *P. teres* isolates were virulent on CI 9819 between 1991 and 1997, but it was still used as an effective resistant donor in some of their breeding programs [31]. Therefore, the nearly equal ratio of virulence and avirulence on this barley line was not expected in Hungary. The two less discriminating differentials, Botond and MV Initium, were bred and produced in Hungary in the past decades. They were susceptible to all tested isolates. Unfortunately, there is no genetic information on the net blotch resistance of Botond and MV Initium. They seem to miss both major genes and quantitative trait loci conferring resistance against the tested isolates at the seedling stage. The finding that none of the barley lines had strong differentiating power in our study emphasizes the need to develop a new differential set for the Hungarian Ptt population.

One IG could be distinguished from the other IGs based on avirulence/virulence on one to five barley lines. Considering the GGE biplot analysis results, the association between IG ‘d’, which included the most virulent Ptt isolates, and the highly resistant barley line CI 11458 suggests non-crossover interaction(s) rather than specific isolate × differential relationships.

There are differences in the compositions of Ptt HCPC clusters and IGs generated in this study. While all the members of the least and most virulent IGs reside in the corresponding HCPC clusters (A and C, respectively), isolates of each of the two remaining IGs, with intermediate virulence, were shared between two HCPC clusters (A or B and B or C). The probable reason for this may be that HCPC clusters and IGs were generated via different data analyses. Jebbouj and El Yousfi [37] had similar observations when they compared HCPC isolate clusters to IGs (pathotypes) determined using mixed model analyses.

Our study also identified barley genotypes that may serve as potential resistance sources against NFNB in Hungary. The seven most resistant differentials had IR scores ≤ 3.9 and were susceptible to 6 to 10 isolates of the 34 tested. CI 11458, the least susceptible line in our study, also showed strong resistance in Iceland [44] and moderate resistance in Australia [41]. CI 5791, our second-least susceptible differential, has also been considered highly resistant against Ptt from several continents [16,41,43,44,46,75]. CI 9825, a generally resistant line with low to moderate susceptibility in Europe [43,44,46], was the third less susceptible to the Hungarian isolates. Twenty-two percent of the fungal isolates were virulent on CI 4207. Virulence frequency on this differential varied from 0 to 100% in Europe according to geographical regions in the 1990s [45], and the Hungarian situation looks similar to what was observed for a Polish region sampled in 1991. Eight and nine of the 34 isolates were virulent on Sylphid and Sebastian, respectively. These two varieties were added to the international differentials because they showed good adult plant resistance against NFNB in cereal breeding stations in Hungary. The results of this study indicate that both possess valuable resistance at the seedling stage, at least against some isolates of the Hungarian Ptt population. Manchurian is known as a moderately resistant/sensitive cultivar [44,46]. It exhibited virulent reactions on ten of the barley lines with an average IR score of 3.9.

## 5. Conclusions

This study characterized the virulence of *Pyrenophora teres* f. *teres,* the causal agent of the net form net blotch disease, on experimental fields of barley in Hungary. The results revealed high and continuous pathogenic variation of the fungus without specific adaptation to the barley differentials tested. In addition, a couple of potential resistance sources against the pathogen’s local isolates were identified, but an extension of the applied differential set, which mostly comprised internationally known barley genotypes, is recommended for future studies on virulence in Hungarian populations of the fungus. These are the first data on the pathogenic variation of *P. teres* f. *teres* in Hungary.

## Figures and Tables

**Table 1:** Summary of the major results on the virulence of *Pyrenophora teres* f. *teres* isolates from the three experimental locations.

Isolate	General Virulence ^1^	Number of Virulent Infections	Pathotype	Virulence Class (Frequency Profile) ^2^				HCPC Cluster ^3^	Isolate Group ^4^
				Avirulent-1	Avirulent-2	Virulent	Highly Virulent		
Karcag, Northern Great Plain of Hungary (Institute for Agricultural Research and Educational Farm of University of Debrecen)									
Ka1	4.16	4	1	3	13	2	2	B	b
Ka2	3.07	2	2	10	8	1	1	A	a
Ka3	3.23	2	2	8	10	0	2	A	b
Ka4	4.74	11	3	2	7	9	2	C	c
Ka5	4.05	6	4	6	8	4	2	A	b
Ka6	4.63	5	5	2	13	2	3	B	b
Ka7	4.79	7	6	1	12	4	3	B	c
Ka8	5.55	10	7	0	10	7	3	B	c
Ka9	4.39	6	8	6	8	3	3	A	a
Ka10	6.54	15	9	0	5	9	6	C	d
Ka11	5.88	11	10	0	9	6	5	B	c
Ka12	4.67	6	11	1	13	3	3	B	c
Ka13	6.47	18	12	0	2	14	4	C	d
Ka14	7.44	20	13	0	0	10	10	C	d
Ka15	5.33	9	14	1	10	6	3	B	c
Ka16	7.33	19	15	0	1	8	11	C	d
Mean of the Isolates from Karcag	5.14 ± 2.29	9.44 ± 5.85							
Kompolt, Northern Hungary (Fleischmann Rudolf Research Institute, Eszterházy Károly University)									
Ko1	2.28	2	4	14	4	2	0	A	a
Ko2	3.41	4	16	9	7	2	2	A	a
Ko3	7.89	20	13	0	0	8	12	C	d
Ko4	5.67	10	17	0	10	5	5	B	c
Ko5	3.96	4	18	5	11	2	2	A	b
Ko6	5.53	9	19	0	11	6	3	B	c
Mean of the Isolates from Kompolt	4.79 ± 2.58	8.17 ± 6.59							
Martonvásár, Central Transdanubia (Agricultural Institute, Centre for Agricultural Research)									
M1	2.17	2	2	13	5	1	1	A	a
M2	3.09	3	20	11	6	2	1	A	b
M3	7.35	15	11	0	5	3	12	C	d
M4	3.19	4	16	10	6	3	1	A	a
M5	6.26	13	22	0	7	8	5	C	d
M6	4.94	7	23	1	12	5	2	B	c
M7	4.47	6	24	1	13	4	2	B	b
M8	5.67	14	25	2	4	10	4	C	c
M9	4.89	7	26	1	12	5	2	B	b
M10	5.15	7	27	0	13	4	3	B	b
M11	3.88	7	26	9	4	4	3	A	b
M12	6.48	17	28	0	3	11	6	C	d
Mean of the Isolates from Martonvásár	4.79 ± 2.47	8.50 ± 4.98							
Overall Mean	4.96 ± 2.41	8.88 ± 5.54							

^1^ The general virulence of an isolate was defined as the mean of the isolate’s IR scores over all the differentials. ^2^ The classes were determined as follows: avirulent-1 = 0–2.49; avirulent-2 = 2.50–4.99; virulent = 5.00–7.49; highly virulent = 7.50–10). ^3^ Identified via hierarchical clustering on the principal components following the correspondence analysis based on the frequency profiles of the isolates. ^4^ Identified via the hierarchical clustering of the isolates using infection response scores.

**Table 2:** Pathotypes (PT) of *Pyrenophora teres* f. *teres* isolates based on the combined avirulence (0) and virulence (1) patterns on 20 barley differentials.

PT	Isolate	Barley Differential																			
		Beecher	Canadian Lake Shore	Corvett	Diamond	Harbin	Manchurian	Prior	Skiff	Tifang	C-20019	CI 5791	CI 4207	CI 9819	CI 9825	CI 11458	Sylphid	Sebastian	Harrington	Botond	MV Initium
1	Ka1	0	0	0	0	0	0	0	1	0	0	0	0	0	0	0	0	0	1	1	1
2	Ka2, Ka3, Ko1, M1	0	0	0	0	0	0	0	0	0	0	0	0	0	0	0	0	0	0	1	1
3	Ka4	1	1	1	1	0	0	0	1	1	1	0	0	1	0	0	0	0	1	1	1
4	Ka5	0	1	0	0	1	0	0	1	0	1	0	0	0	0	0	0	0	0	1	1
5	Ka6	0	1	0	0	1	0	0	0	0	0	0	0	0	0	0	0	1	0	1	1
6	Ka7	0	0	0	0	1	0	1	0	1	0	0	0	1	0	0	0	0	1	1	1
7	Ka8	1	1	1	0	1	0	1	0	0	1	0	0	1	0	0	0	0	1	1	1
8	Ka9	0	0	1	1	0	0	0	0	0	1	0	0	0	0	0	0	0	1	1	1
9	Ka10	0	1	1	1	1	1	1	1	1	1	0	1	1	0	1	0	1	0	1	1
10	Ka11	0	1	1	0	1	1	1	0	0	1	1	0	1	0	0	0	0	1	1	1
11	Ka12	1	0	1	0	0	0	1	0	0	0	0	0	1	0	0	0	0	0	1	1
12	Ka13	1	1	0	1	1	1	1	0	1	1	1	1	1	1	1	1	1	1	1	1
13	Ka14, Ko3	1	1	1	1	1	1	1	1	1	1	1	1	1	1	1	1	1	1	1	1
14	Ka15	0	0	1	0	1	0	0	1	0	1	0	0	1	1	0	1	0	0	1	1
15	Ka16	1	1	1	1	1	1	1	1	1	0	1	1	1	1	1	1	1	1	1	1
16	Ko2, M4	0	0	1	0	0	0	0	0	0	0	0	0	0	0	0	0	0	1	1	1
17	Ko4	0	1	1	1	1	0	0	1	1	1	0	0	1	0	0	0	0	0	1	1
18	Ko5	0	0	0	0	0	0	0	1	0	0	0	0	1	0	0	0	0	0	1	1
19	Ko6	1	0	1	1	1	0	0	1	0	1	0	0	1	0	0	0	0	0	1	1
20	M2	0	0	0	0	1	0	0	0	0	0	0	0	0	0	0	0	0	0	1	1
21	M3	1	1	1	1	1	0	1	1	1	1	1	0	1	0	1	0	0	1	1	1
22	M5	0	0	1	1	1	1	0	1	1	1	0	1	1	0	1	0	0	1	1	1
23	M6	0	1	1	0	1	1	0	0	0	0	0	1	0	0	0	0	0	0	1	1
24	M7	0	1	1	0	1	0	0	1	0	0	0	0	0	0	0	0	0	0	1	1
25	M8	0	1	1	1	1	1	0	0	1	1	1	1	1	1	0	0	0	1	1	1
26	M9, M11	0	1	0	0	1	0	0	1	0	0	0	0	0	0	0	1	1	0	1	1
27	M10	1	0	1	0	0	0	0	1	0	0	0	0	0	0	0	1	0	1	1	1
28	M12	0	1	1	1	1	1	1	1	1	1	1	1	1	0	1	0	1	1	1	1
Total no. of PTs		9	16	19	12	20	9	10	16	11	15	7	8	17	5	7	6	7	15	28	28

**Table 3:** Virulence (1) and avirulence (0) of the *Pyrenophora teres* f. *teres* isolate groups on five barley differentials.

Isolate Group	Barley Differential				
	Diamond	Harbin	Tifang	CI 9819	CI 11458
IG ‘a’	1/0	0	0	0	0
IG ‘b’	0	1/0	0	1/0	0
IG ‘c’	1/0	1/0	1/0	1/0	0
IG ‘d’	1	1	1	1	1

## Data Availability

Data are contained within the article and Supplementary Materials.

## Supplementary Materials

The following supporting information can be downloaded at: https://www.mdpi.com/article/10.3390/jof10030184/s1, Table S1: Origin of *Pyrenophora teres* f. *teres* isolates and the infection response scores obtained on 20 barley differentials.
