# Supplementary material for: Virulence Spectra of Hungarian Pyrenophora teres f. teres Isolates Collected from Experimental Fields Show Continuous Variation without Specific Isolate × Barley Differential Interactions
Source: J Fungi (Basel). 2024 Feb 28;10(3):184. doi: 10.3390/jof10030184 (PMC10971109; doi:10.3390/jof10030184)
Supplement: Supplementary file 1 [file jof-10-00184-s001.zip › jof-2807912-supplementary.pdf]

Table S1. Origin of *Pyrenophora teres* f. *teres* isolates and the infection response scores [1] obtained on 20 barley differentials.

**Iso-late**	**Cultivar**	**Year**	**Barley differential**																			
			**Beecher**	**Canadian Lake Shore**	**Corvett**	**Diamond**	**Harbin**	**Manchurian**	**Prior**	**Skiff**	**Tifang**	**C-20019**	**CI 5791**	**CI 4207**	**CI 9819**	**CI 9825**	**CI 11458**	**Sylphid**	**Sebastian**	**Harrington**	**Botond**	**MV Initium**
Karcag, Northern Great Plain of Hungary (Institute for Agricultural Research and Educational Farm of University of Debrecen)																						
Ka1	KG Puszta	2017	2.25	4.85	4.00	2.90	3.80	1.90	4.40	**5.50**	2.90	4.85	4.10	3.20	3.20	3.70	1.40	3.55	4.50	**5.10**	**7.90**	**9.20**
Ka2	KG Apavár	2017	1.60	1.90	4.80	3.10	4.65	3.00	3.90	1.10	4.00	2.15	1.25	1.50	4.10	1.60	1.25	0.90	1.70	4.80	**7.50**	**6.60**
Ka3	KH Tas	2017	2.35	2.15	3.65	1.95	2.55	1.35	2.40	4.15	2.00	3.40	2.75	2.30	2.55	2.85	1.00	2.85	4.15	4.25	**8.00**	**7.90**
Ka4	KG Puszta	2018	**5.15**	**5.50**	**5.20**	**5.50**	3.75	3.90	3.00	**5.70**	**5.35**	**5.50**	3.00	3.70	**5.00**	3.10	1.80	3.00	1.90	**5.20**	**9.60**	**9.90**
Ka5	KG Konta	2018	2.65	**6.45**	2.40	2.90	**7.20**	2.10	1.55	**6.00**	3.10	**5.00**	1.45	2.30	3.55	2.70	3.40	4.35	2.00	2.90	**9.60**	**9.40**
Ka6	KG Apavár	2018	4.30	**9.90**	3.70	3.80	**7.65**	3.00	2.60	2.35	4.20	4.65	3.30	4.30	4.75	3.90	2.80	3.65	**5.20**	2.20	**6.75**	**9.50**
Ka7	KH Tas	2018	3.70	4.60	3.85	4.55	**7.95**	2.55	**7.10**	3.60	**5.00**	4.00	4.20	4.40	**7.90**	3.45	1.40	3.00	3.70	**5.90**	**6.30**	**8.60**
Ka8	KH Kárpátia	2018	**7.10**	**7.00**	**6.60**	4.70	**8.05**	4.70	**5.30**	4.90	4.80	**5.30**	3.00	4.20	**6.10**	2.80	3.40	4.85	4.45	**5.10**	**8.70**	**10.00**
Ka9	KH Anatólia	2018	4.80	1.80	**9.40**	**5.30**	2.00	2.20	3.20	4.10	4.60	**6.20**	1.80	3.40	4.30	2.00	3.40	1.90	2.80	**7.40**	**7.50**	**9.70**
Ka10	KH Rudolf	2018	3.10	**6.60**	**6.30**	**7.60**	**7.45**	**6.15**	**5.00**	**9.00**	**7.40**	**7.25**	4.65	**5.00**	**9.50**	3.30	**9.60**	4.65	**7.00**	4.50	**8.90**	**7.90**
Ka11	Patina	2018	4.55	**7.90**	**7.15**	4.90	**9.25**	**5.00**	**7.10**	4.35	4.40	**5.45**	**5.00**	3.55	**8.20**	4.30	3.40	4.15	4.30	**5.40**	**9.50**	**9.80**
Ka12	Boreale	2018	**5.10**	3.90	**5.25**	3.80	3.85	3.30	**7.50**	4.65	3.80	4.60	4.15	3.10	**7.65**	3.20	2.35	3.80	4.00	4.40	**8.00**	**7.00**
Ka13	Boreale	2018	**6.80**	**5.90**	4.20	**7.70**	**6.05**	**5.20**	**8.30**	4.50	**6.10**	**7.00**	**6.65**	**5.50**	**6.00**	**6.65**	**7.30**	**5.00**	**6.10**	**6.90**	**9.00**	**8.60**
Ka14	MV Initiun	2018	**7.50**	**5.00**	**9.60**	**7.00**	**6.10**	**5.50**	**9.75**	**8.70**	**5.90**	**9.85**	**7.20**	**5.10**	**8.70**	**6.60**	**7.85**	**6.75**	**5.15**	**8.40**	**9.20**	**8.90**
Ka15	GKH 30-15	2018	4.75	4.30	**5.00**	4.15	**5.95**	4.20	4.00	**7.20**	4.55	**9.65**	3.90	3.60	**5.25**	**5.10**	2.45	**5.15**	4.15	4.10	**9.60**	**9.50**
Ka16	Faktor	2018	**7.10**	**7.50**	**7.25**	**8.40**	**7.55**	**8.00**	**9.15**	**5.15**	**8.10**	4.75	**5.60**	**7.50**	**9.20**	**6.00**	**6.85**	**5.00**	**5.00**	**9.45**	**9.60**	**9.40**
Kompolt, Northern Hungary (Fleischmann Rudolf Research Institute, Eszterházy Károly University)																						
Ko1	MV Initium	2017	2.30	1.20	3.00	2.20	1.00	1.40	1.50	3.10	2.60	2.00	1.00	1.60	2.00	1.20	1.10	1.00	1.20	3.50	**5.90**	**6.70**
Ko2	KG Apavár	2018	2.45	2.60	**7.15**	3.70	1.55	2.20	1.45	3.15	2.80	4.00	1.70	2.00	3.00	2.90	0.90	2.00	1.00	**6.70**	**7.85**	**9.10**
Ko3	KG Apavár	2018	**5.50**	**8.50**	**9.10**	**10.00**	**8.05**	**5.90**	**9.10**	**8.30**	**9.40**	**9.25**	**6.40**	**5.40**	**9.70**	**7.10**	**9.00**	**6.60**	**5.65**	**6.50**	**9.70**	**8.60**

Table S1. *Cont.*

Isolate	Cultivar	Year	Barley differential																			
			**Beecher**	**Canadian Lake Shore**	**Corvett**	**Diamond**	**Harbin**	**Manchurian**	**Prior**	**Skiff**	**Tifang**	**C-20019**	**CI 5791**	**CI 4207**	**CI 9819**	**CI 9825**	**CI 11458**	**Sylphid**	**Sebastian**	**Harrington**	**Botond**	**MV Initium**
Ko4	KWS	2018	4.75	**7.10**	**8.00**	**5.00**	**9.20**	4.30	4.45	**5.00**	**5.75**	**5.15**	4.80	4.50	**8.15**	4.50	3.60	4.40	4.15	3.80	**8.10**	**8.60**
Ko5	Faktor	2018	2.50	4.90	1.30	4.80	4.15	2.30	4.85	**5.00**	2.70	3.15	2.40	3.10	**6.20**	3.30	1.90	4.40	2.00	4.00	**8.65**	**7.50**
Ko6	Boreale	2018	**5.50**	4.40	**5.50**	**5.50**	**6.20**	4.45	4.40	**5.15**	4.90	**7.15**	4.40	4.30	**8.35**	4.35	3.60	3.60	4.50	4.60	**9.90**	**9.90**
Martonvásár, Central Transdanubia (Agricultural Institute, Centre for Agricultural Research)																						
M1	KH Turul	2008	4.45	0.60	1.45	3.00	0.50	0.40	0.25	3.90	2.40	2.75	1.20	0.30	2.40	1.25	0.00	1.00	0.00	2.90	**6.20**	**8.40**
M2	KH Center	2008	3.13	4.80	3.50	2.00	**6.69**	2.20	1.43	2.14	2.40	2.50	2.00	2.00	3.14	1.14	1.30	3.00	1.00	2.40	**7.60**	**7.40**
M3	KH Kárpátia	2018	**5.20**	**9.00**	**8.75**	**9.00**	**8.40**	4.00	**9.90**	**9.00**	**8.70**	**8.80**	**5.15**	3.10	**9.65**	4.25	**6.10**	4.85	4.75	**9.70**	**9.55**	**9.20**
M4	KG Konta	2018	2.10	1.45	**5.15**	2.60	2.15	2.00	3.15	1.10	3.10	3.10	1.80	1.60	4.00	3.20	1.60	2.10	2.20	**5.90**	**6.70**	**8.70**
M5	KH Zsombor	2018	4.00	4.50	**9.15**	**5.90**	**6.85**	**6.70**	3.65	**6.40**	**6.35**	**5.70**	4.40	**6.50**	**9.75**	4.20	**6.65**	4.85	4.90	**7.60**	**8.30**	**8.80**
M6	MV Initium	2018	4.90	**7.00**	**6.50**	3.60	**7.40**	**5.60**	4.00	4.60	4.00	4.90	1.90	**5.10**	3.90	2.70	2.65	3.90	3.15	4.45	**9.30**	**9.20**
M7	Faktor	2018	3.30	**6.60**	**5.00**	3.25	**6.30**	4.30	2.10	**5.90**	3.30	3.65	3.20	3.10	3.30	2.70	3.00	3.70	4.40	4.30	**9.30**	**8.70**
M8	KWS Meridian	2018	3.70	**5.75**	**6.50**	**6.00**	**6.55**	**6.00**	4.85	4.90	**7.65**	**5.25**	**6.20**	**7.00**	**8.20**	**5.15**	1.10	3.00	2.25	**6.50**	**8.00**	**8.80**
M9	Siberia	2018	3.65	**6.90**	4.00	3.90	**6.30**	4.30	2.45	**7.45**	4.15	4.00	2.90	4.15	4.35	2.55	4.30	**5.00**	**5.90**	4.10	**8.25**	**9.20**
M10	GKH 30-15	2018	**5.20**	4.80	**6.00**	4.50	4.65	4.90	3.55	**8.45**	4.00	4.55	3.45	3.80	4.65	3.65	4.00	**5.30**	4.15	**5.00**	**9.60**	**8.70**
M11	Boreale	2018	2.50	**5.00**	2.35	1.60	**5.55**	2.00	1.25	**9.80**	1.90	3.90	1.00	1.60	2.25	2.60	4.15	**5.10**	**6.90**	2.00	**7.80**	**8.30**
M12	KH TAS	2018	3.15	**5.50**	**8.75**	**5.90**	**6.95**	**7.50**	**5.35**	**7.50**	**6.45**	**5.65**	**5.35**	**6.60**	**8.80**	4.30	**5.80**	4.75	**5.80**	**6.40**	**9.15**	**10.00**
Overall mean			4.15	5.17	5.57	4.73	5.65	3.90	4.47	5.35	4.67	5.15	3.57	3.78	5.82	3.60	3.54	3.86	3.82	5.19	8.40	8.76

[1] Bold numbers indicate virulence (≥ 5.00) according to Tekauz [55].
